# Possible Dissemination of *Escherichia* co*li* Sequence Type 410 Closely Related to B4/H24RxC in Ghana

**DOI:** 10.3389/fmicb.2021.770130

**Published:** 2021-12-01

**Authors:** Samiratu Mahazu, Isaac Prah, Alafate Ayibieke, Wakana Sato, Takaya Hayashi, Toshihiko Suzuki, Shiroh Iwanaga, Anthony Ablordey, Ryoichi Saito

**Affiliations:** ^1^Department of Molecular Microbiology, Tokyo Medical and Dental University, Tokyo, Japan; ^2^Department of Environmental Parasitology, Tokyo Medical and Dental University, Tokyo, Japan; ^3^Department of Molecular Virology, Tokyo Medical and Dental University, Tokyo, Japan; ^4^Department of Bacterial Pathogenesis, Tokyo Medical and Dental University, Tokyo, Japan; ^5^Department of Bacteriology, Noguchi Memorial Institute for Medical Research, University of Ghana, Accra, Ghana

**Keywords:** Extra-intestinal pathogenic *E. coli* (ExPEC), ST410, B3/H24Rx, B4H24RxC, Oxacillinase-181 (OXA-181)

## Abstract

Extra-intestinal pathogenic *Escherichia coli* (ExPEC) is one of the world’s leading causes of bloodstream infections with high mortality. Sequence type 410 (ST410) is an emerging ExPEC clone resistant to a wide range of antibiotics. In this study, we investigated the epidemiology of 21 ST410 *E. coli* isolates from two Ghanaian hospitals. We also investigated the isolates within a global context to provide further insight into the dissemination of this highly pathogenic clone. A phylogenetic tree of the 21 isolate genomes, along with 102 others from global collection, was constructed representing the ensuing clades and sub-clades of the ST: A/H53, B2/H24R, B3/H24Rx, and B4/H24RxC. The carbapenem-resistant sub-clade B4/H24RxC is reported to have emerged in the early 2000s when ST410 acquired an IncX3 plasmid carrying a *bla*_OXA–__181_ carbapenemase gene, and a second carbapenemase gene, *bla*_NDM–__5_, on a conserved IncFII plasmid in 2014. We identified, in this study, one *bla*_OXA–__181_–carrying isolate belonging to B4/H24RxC sub-lineage and one carrying *bla*_NDM–__1_ belonging to sub-lineage B3/H24Rx. The *bla*_OXA–__181_ gene was found on a 51kb IncX3 plasmid; pEc1079_3. The majority (12/21) of our Ghanaian isolates were clustered with international strains described by previous authors as closely related strains to B4/H24RxC. Six others were clustered among the ESBL-associated sub-lineage B3/H24Rx and three with the globally disseminated sub-lineage B4/H24RxC. The results show that this highly pathogenic clone is disseminated in Ghana and, given its ability to transmit between hosts, it poses a serious threat and should be monitored closely.

## Introduction

The World Health Organization (WHO) in its 2014 report hinted of a looming medical health crisis on account of the rapid evolution and dissemination of antibiotic resistant strains. Today, the proliferation of such clones and infections they cause outpace the potent antibiotics available, undermining the many advances that have been made so far in modern medicine (World Health Organization [WHO], 2014).

*Escherichia coli* sequence type 410 (ST410) is an emerging multidrug-resistant pathogenic clone that belongs to a group of strains called extra-intestinal pathogenic *E. coli* (ExPEC), responsible for the wide spectrum of invasive extra-intestinal infections (Roer et al., 2018). It is known to belong to phylogroup A, a group thought to comprise mildly virulent commensal bacteria, and is possibly the reason why ST410 has not attained global prominence (Falgenhauer et al., 2016; Roer et al., 2018). However, a recent study found ST410 to be an originator of clonal complex 23, belonging to a phylogroup in between A and B1 (Falgenhauer et al., 2016). Further evidence has shown that bacteria in this phylogroup can in fact, cause life-threating infections such as sepsis, and capable of elaborating different virulence factors (Chakraborty et al., 2015; Falgenhauer et al., 2016). Molecular epidemiological studies classify ST410 as a host-generalist with enhanced colonization abilities that make it persist in its hosts for a long time (Falgenhauer et al., 2016; Schaufler et al., 2016; Roer et al., 2018). A specific, recombination-driven, carbapenem-resistant clone, B4/H24RxC with the plasmid IncX3 carrying either *bla*_OXA–__181_ or *bla*_NDM–__5_ is reportedly disseminating globally. The IncX3 plasmid has been shown to be constantly exchanging either *bla*_OXA–__181_ or *bla*_NDM–__5_ without any ramification to the plasmid stability or fitness (Feng et al., 2019). In Northern America, Europe, and Southeast Asia, this clone appears to be prevalent, in addition to a fluoroquinolone- and extended-spectrum cephalosporin–resistant sub-clade B3/H24Rx (Nadimpalli et al., 2019).

The rapid dissemination of antimicrobial resistance has been facilitated mainly by extended spectrum beta lactamases (ESBLs) through clonal expansion and/or plasmid transfer (Schaufler et al., 2016). Of all the ESBL genes described thus far, CTX-M-15 is the most reported in infections involving ExPEC in Africa and worldwide (Falgenhauer et al., 2016; Irenge et al., 2019). We have also recently reported carbapenem-resistant ST410 isolates among Ghanaian isolates (Ayibieke et al., 2018; Prah et al., 2021). In response to this global crisis, the WHO instituted a global action plan to mitigate the problem of antimicrobial resistance, with one key measure put in place being antibiotic stewardship through effective surveillance programs to help prevent the spread of resistant strains.

Despite the progress made in phenotypic identification of resistant strains, to date, there are only a handful of data on genomic characterization of ExPEC strains, particularly in the African region including Ghana, making it almost unfeasible to construe the mechanisms underlining their dissemination to control their spread. Therefore, we investigated 21 ESBL-producing *E. coli* ST410 isolates from Ghana using whole-genome sequencing (WGS) and explored their phylogenetic relationships with 102 GenBank genomes collected from other regions of the world. We also analyzed the antibiotic resistance and virulence markers as well as the plasmid replicon sequence types in the genomes.

## Materials and Methods

### Strain Isolation, Identification, and Antimicrobial Susceptibility Testing

Twenty-one *E. coli* ST410 isolates were obtained from an amplest collection of 164 *E*. *coli* isolates recovered from clinical specimens at the Effia Nkwanta Regional Hospital and Tamale Teaching Hospital between March 2015 and April 2016. The isolates were stored in 10% skimmed milk at −80°C prior to use. The clinical specimens included urine (*n* = 10), high vaginal swab (*n* = 1), pus (= 1), sputum (*n* = 1), stool (*n* = 1), and others (*n* = 7). These isolates were identified using a MALDI Biotyper (Bruker Daltonics, Karlsruhe, Germany). The isolates were pre-characterized by ESBL production and then, multi-locus sequence typing (MLST) of ESBL producers. MLST grouped the ESBL isolates into 20 sequence types with *E. coli* ST131 (*n* = 24, 23.5%), ST410 (*n* = 21, 20.6%) and ST617 (*n* = 19, 18.6%) constituting about three-fifths of the total number. However, this study focused on isolates identified as ST410. The isolation sites of the 21 *E. coli* isolates are presented in Supplementary Data Sheet 1.

Antibiogram profiles of the isolates to 16 antibiotics were determined by minimum inhibitory concentration (MIC) as previously described (Prah et al., 2021). The MICs of cefmetazole and ciprofloxacin were determined by the agar dilution method. The results were interpreted according to the guidelines of the Clinical Laboratory Standards Institute (CLSI) M100-S30. *E. coli* reference strain, ATCC 25922 was used as a negative quality control strain. ESBL phenotypes were confirmed using *Klebsiella pneumoniae* ATCC 700603 as a quality control strain.

### Characterization of Extended Spectrum Beta Lactamases Genes and Multi-Locus Sequence Typing of Extended Spectrum Beta Lactamases Producers

DNA was extracted using CICA Geneus DNA Extraction Reagent (Kanto Chemical Co., Tokyo, Japan). Briefly, a bacterial suspension equivalent to McFarland no. 3 turbidity was prepared in a 1 mL volume. Reagents A and B (provided in the kit) were mixed in a ratio of 1:10. One microliter of the bacterial suspension was added to the reagent. The reaction was incubated at 72°C for 6min and 94°C for 3 min in a thermal cycler in total reaction volume of 25 μl. The resulting reaction mixture was used as template for PCR.

Beta-lactamase–encoding genes in ESBL producers were screened for, as previously described (Dallenne et al., 2010). We screened for *bla*_CTX–M–__15_ following the protocol described by Moghanni et al. (2018). PCR was conducted using EmeraldAmp Max PCR Mastermix (2× Premix) (Takara, Tokyo, Japan) in a total reaction volume of 10 μl. One microliter of the DNA template was used for the reaction. PCR products were separated on agarose gel by electrophoresis. Band sizes (*bla*_*TEM*_-800 bp, *bla*_SHV_-713 bp, *bla*_CTX–M_-688 bp, *bla*_OXA–__1__–like_-564 bp, *bla*_CTX–M–__15_-875 bp) were estimated with the aid of a 100 bp molecular marker. *bla*_CTX–M–__14_ and *bla*_CTX–M–__27_ were distinguished using PCR and subsequently, sanger sequencing using primer sets *bla*_CTX–M–group__9__–F_ (5′-ATGGTGACAAAGAGAGTGCAA-3′) and *bla*_CTX–M_group9-R (5′-CCCTTCGGCGATGATTCT-3) on 3730xl DNA Analyzer (Thermo Fisher Scientific) with BigDye Terminator v3.1 Cycle Sequencing Kit (Thermo Fisher Scientific).

PCR amplification was carried out with an initial denaturation at 94°C for 4 min, followed by 30 cycles of 94°C for 1 min, 55°C for 1 min, and 72°C for 1 min. The amplified DNA sequences were then sequenced and compared with those in the National Center for Biotechnology Information database using the BLAST program. Multi-locus sequence typing of all ESBL isolates was performed as described by Lau et al. (2008). The amplified fragments had an allele number allocated according to EnteroBase^1^. Based on the allelic profiles at the seven loci, STs were assigned.

### Whole-Genome Sequencing and Assembly

Genomic DNA was extracted from all identified ST410 isolates for short read sequencing using a Nucleospin Tissue Kit (Macherey-Nagel, Düren, Germany). The purity (1.8–2.0) and concentration of DNA were checked using Nanodrop Lite spectrophotometer and Invitrogen Qubit 4 fluorometer (Thermo Fisher Scientific). DNA library was prepared using the MGIEasy FS PCR-Free DNA Library Prep set (MGI, Cat. No. 1000013454) according to the manufacturer’s instructions. DNA (400 ng) was fragmented by enzymatic fragmentation (target size 400–660 bp) using magnetic beads in an 8-min reaction and subjected to a 2-step size selection with an insert size of about 400 bp. DNA end-repair and adapter ligation were conducted using the MGIEasy PF Adapters-96 Kit. After heat-denaturing the adapter-attached DNA, it was circularized into ssDNA. A total of 75 fmol of DNA nanoballs, with a concentration of 17.5 ng/μl. was prepared by combining 50 samples of ssDNA in equivalent amounts. The barcoded library was then loaded onto a DNBSEQ Flow cell ID F300000969 and sequenced on MGI DNBSEQ-G400FAST (MGI Tech Co., Shenzhen, China) for short reads. Low-quality reads (≤ Q30), short reads (≤ 10 bp), and accidental residual adapter sequences were filtered out. For long read sequencing, DNA was extracted using Magattract HMW DNA kit (Qiagen, Hildon, Germany). Two isolates labeled Ec45 and Ec1079 were further sequenced on Nanopore MinION (Oxford Nanopore Technologies, Oxford, UK). DNA libraries were prepared using the ligation sequence kit SQK-LSK109 (target fragment size > 3kb) and sequencing was performed on a Flongle Flow Cell (Oxford Nanopore Technologies, Oxford, UK). Low-quality reads (≤ Q10) and long reads (≤ 1000 bp) were filtered out. Using Unicycler v0.4.8 with de Brujin graph, a *de novo* assembly was run to generate a draft genome sequence for each isolate. The number of contigs obtained for all samples was 100–200. Hybrid assemblies using reads from DNBSEQ-G400FAST (short reads) and Nanopore (long reads) were constructed to generate finished genomes of strains Ec45 and Ec1079. The sequences generated by Unicycler were used for all analyses. Details of sequencing and assembly are attached as Supplementary Data Sheet 3. Sequences were annotated using the online tools RAST^2^ and BLAST searches.

### Phylogenesis, Bioinformatic Analysis, and Genetic Diversity

To get a broader understanding of the dissemination of ST410, we constructed a maximum-likelihood phylogenetic tree of all 21 isolates along with 102 international strains retrieved from GenBank (last accessed February 2021). The raw reads were assembled using the Unicycler v0.4.8. Assembled genomes were annotated using prokka v1.13 (Seemann, 2014). The pangenome analysis software, roary v3.20.0 (Page et al., 2015), was used to determine the number of core and accessory genes present in the genomes of the isolates. All genome sequences were mapped onto a reference genome (accession no: CP034958) and variants were called using snippyv3.1 (Seemann, 2007). The core single nucleotide polymorphisms (SNPs) alignment generated was cleaned using integrated scripts within snippy. Gubbinsv2.4.1 (Croucher et al., 2015) was used to remove recombinant regions. SNP-Sites (Page et al., 2016) was used to extract SNPs from the recombination-free, cleaned file. The length of core-genome SNP alignment generated was 3077 bp. A SNPs-based recombination-free, phylogenetic tree was then inferred using iqtree (Nguyen et al., 2015) and visualized using iTOL v6.1.1 (Letunic and Bork, 2019).

To estimate how our ST410 strains are genetically related to themselves, and to the other isolates, we constructed a core genome alignment-based minimum spanning tree using PHYLOViZ online^3^.

Acquired resistance genes and chromosomal point mutations were identified by resfinder v4.0 (Bortolaia et al., 2020). Insertion sequences were identified using ISFinder^4^. FimTyper^5^ was used to assign fim types. Plasmid replicon types were determined using PlasmidFinder^6^. IncF plasmid replicons were further subtyped using the pMLST^7^. Virulence genes were characterized *in silico* at 90% threshold and 60% minimum length using^8^, all available at the center for genomic epidemiology database.

The genetic environment surrounding *bla*_OXA–__181_ was investigated. Plasmids sequences were compared using the BLAST Ring Image Generator tool (BRIG) (Alikhan et al., 2011). Complete genomes of strains Ec45 and Ec1079 and raw read sequences of the other 19 *E. coli* strains have been deposited in GenBank under the Bioproject PRJNA473419. Accession numbers of all strains used in this study can be found in Supplementary Data Sheet 1.

## Results

### Antibiotic Susceptibility Profiles and Extended Spectrum Beta Lactamases, Sequence Types, and *fim*H Alleles

High rate (100%) of resistance was observed to eight antibiotics representing four antibiotic classes (penicillin, cephalosporins, sulfonamides, and quinolones). The lowest resistance rate found was to amikacin (9.5%). The rates of antibiotic resistance and MIC_50_ and MIC_90_ values of all isolates are shown in Table 1. All isolates (*n* = 21, 100%) were resistant to cefotaxime and confirmed as ESBL-producers. ESBL gene *bla*_CTX–M–__15_ was detected in all the strains. Multi-locus sequence typing determined the 21 isolates as belonging to ST410 with their characteristic *fim*H24 alleles. Except for two from the United States that had *fim*H53, all other genomes included had *fim*H24 (Supplementary Data Sheet 1).

**TABLE 1:** Minimum inhibitory concentration50 (MIC_50_) and Minimum inhibitory concentration90 (MIC_90_) values and antibiotics resistance percentage values of *E. coli* ST410 isolates.

**Antimicrobial agents**	**Breakpoint for resistance (μg/mL)**	**% resistance**	**MIC (μg/mL)**		
			**Range**	**MIC_50_**	**MIC_90_**
Piperacillin	≥128	100.0	>64–>64	>64	>64
Cefazolin	≥8	100.0	>16–>16	>16	>16
Cefotaxime	≥4	100.0	32–>32	>32	>32
Ceftazidime	≥16	100.0	16–>16	>16	>16
Cefepime	≥16	95.2	8–>16	>16	>16
Sulbactam/Ampicillin	≥16/32	85.7	≤8/16–>8/16	>8/16	>8/16
Cefpodoxime	≥8	100.0	≤4–>4	>4	>4
Aztreonam	≥16	95.2	≤0.5–>16	>16	>16
Imipenem	≥4	0.0	≤0.25–2	≤0.25	≤0.25
Meropenem	≥4	0.0	≤0.25–1	≤0.25	≤0.25
Gentamicin	≥16	81.0	0.5–>8	>8	>8
Amikacin	≥64	9.5	2–>32	4	32
Minocycline	≥16	71.4	1–> 8	>8	>8
Fosfomycin	≥256	0.0	≤32– ≤32	≤32	≤32
Sulfamethoxazole/Trimethoprim	≥76/4	100.0	>38/2–>38/2	>38/2	>38/2
Levofloxacin	≥2	100.0	>4–>4	>4	>4
Cefmetazole	≥64	14.3	1–128	2	64
Ciprofloxacin	≥1	100.0	>8–>8	>8	>8

### Whole-Genome Sequencing, Phylogenetic Analysis, and Genetic Diversity

**FIGURE 1:**
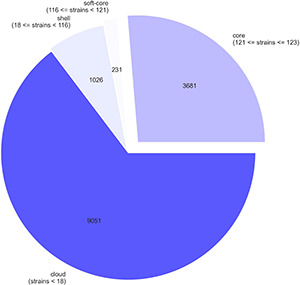
A pie chart representation of pangenome of 123 *E. coli* strains. The chart shows the proportion of core and accessory genes present in the strains.

**FIGURE 2:**
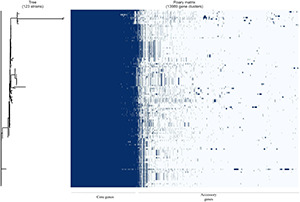
Gene presence/absence matrix from pangenome analysis of 123 *E. coli* isolates with Roary and roary.plots. Each row shows gene profile of each isolate.

Pangenome analysis of the 21 Ghanaian strains generated a total of 6840 genes, with 4112 core genes in 99–100% of the strains. Seventy-three soft core genes were found in 95–99% of the strains, 839 shell genes in 15–95%, and 1816 cloud genes in 0–15%. These results are presented in Supplementary Figure 1. Gene presence and absence matrix generated for the 21 strains is shown in Supplementary Figure 2. Results of pangenome analysis of all 123 genomes are shown in Figures 1, 2 in the main text.

The most prevalent resistance genes identified in the genomes of our isolates included *aac(6′)-Ib-cr* (fluoroquinolone resistance); *aac(6′)-Ib-cr*, *aph(6)-Id*, and *aadA5* (aminoglycoside resistance);*mph(A)* (macrolide resistance); *sul1* and *sul2* (sulfonamide resistance); and *bla*_*CMY–*__2_ (AmpC resistance) among others (Supplementary Data Sheet 1). In all 21 isolates, there were amino acid substitutions in the quinolone resistance–determining regions (QRDRs) of ParC (S80I), ParcE (S458A), and GyrA (S83L, D87N) that conferred high level of resistance to fluoroquinolones (Supplementary Data Sheet 1). Of the 102 genomes included, 92 also had amino acid substitutions in the QRDRs of ParC (S80I), ParcE (S458A), and GyrA (S83L, D87N). Two strains also carried carbapenemase genes: one with *bla*_OXA–__181_ and the other *bla*_NDM–__1_.

IncF replicon types IncFIA and IncFIB were repeatedly found in all 21 of the strains. Notably, the plasmid type IncX3, which is commonly associated with carbapenem-resistant ST410 strains was found in only one strain and in 25 of the 102 other genomes included (Supplementary Data Sheet 1). Six different IncF plasmid multi-locus sequence types (pMLST) were identified, namely, pMLST F22:A4:B1 (*n* = 2), F31:A4:B1 (*n* = 5), F36:A4:B1 (*n* = 5), F105:A4:B1 (*n* = 4), F1:A1:B49 (*n* = 4), and F-:A1:B1 (*n* = 1). *bla*_OXA–__181_- and *bla*_NDM–__1_-producing isolates harbored F1:A1:B49.

All 21 isolates were examined for the presence of ExPEC virulence-associated markers, and multiple were found in more than 70% of the isolates. They contained genes encoding heat-resistant agglutinin (*hra*), iron transport protein (*sitA*), cytotoxic necrotizing factors (*cnf*1), aerobactin (*iut*A), yersiniabactin (*fyu*A), P-fimbriae (*pap*A and *pap*C), and serum resistance (*tra*T). The full complement of virulence genes is available in Supplementary Data Sheet 2.

**FIGURE 3:**
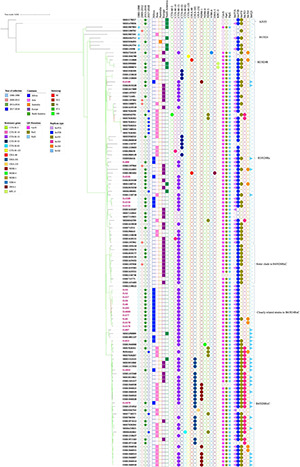
Maximum-likelihood phylogeny of 123 *Escherichia coli* ST410 strains, 21 (labeled in pink) from Ghana, and 102 global collections sourced from Genbank. The phylogenetic tree was constructed based on single-nucleotide polymorphisms alignment of the core genomes after filtering out recombinant sites. Branch patterns were assessed by 1,000 bootstrap replicates. Bootstrap support values ranging from 90 to 100 are displayed in colors on the branches of the tree. Red color shows minimum and lemon green color shows maximum. Tree shows sub-lineages described within ST410, namely, A/H53, B1/H24, B2/H24R, B3/H24Rx, the sister clade to B4/H24RxC, closely related strains to B4/H24RxC and B4/H24RxC. The year and continents of isolation, resistance genes, QR mutations and plasmid replicon types are indicated by colored regions. Strain IDs of 21 Ghanaian isolates and accession numbers of the other 102 strains are labeled at the tips.

In investigating the 21 Ghanaian strains within the global context, 102 other genomes were included. These originated from five continents covering 29 countries: China (15), Philippines (13), Germany (11), United States (11), Netherlands (8), Thailand (8), United Kingdom (5), Denmark (3), Belgium (2), Romania (2), Laos (2), Norway (2), Singapore (2), Vietnam (2), Australia (1), Malawi (1), Czech Republic (1), Hungary (1), Ireland (1), Mexico (1), Nepal (1), Poland (1), Saudi Arabia (1), Spain (1), Tanzania (1), Unknown African country (1), and Turkey (1); three with no information on their country of origin were also included. Phylogenetic framework clustered all 123 strains into five known sub-lineages: A/H53, B1/H24, B2/H24R, B3/H24Rx, B4/H24RxC, and two newly described (Feng et al., 2019) B4/H24RxC sublineages (sister clade to B4/H24RxC and closely related strains to B4/H24RxC). In all, only two strains belonged to A/H53. Sub-lineages B1/H24 and B2/H24R comprised seven and six strains respectively. B3/H24Rx was composed of 47 strains and while eleven strains clustered as sister clades to B4/H24RxC, fifteen strains clustered as closely related strains to B4/H24RxC. Thirty-five strains constituted sub-lineage B4/H24RxC (Figure 3). With regards to Ghanaian strains, most of the Ghanaian isolates (12/21) clustered closely with the sub-lineage described as closely related to B4/H24RxC. Six strains were clustered with the B3/H24Rx sub-lineage and three were clustered among the B4/H24HRxC sub-lineage.

**FIGURE 4:**
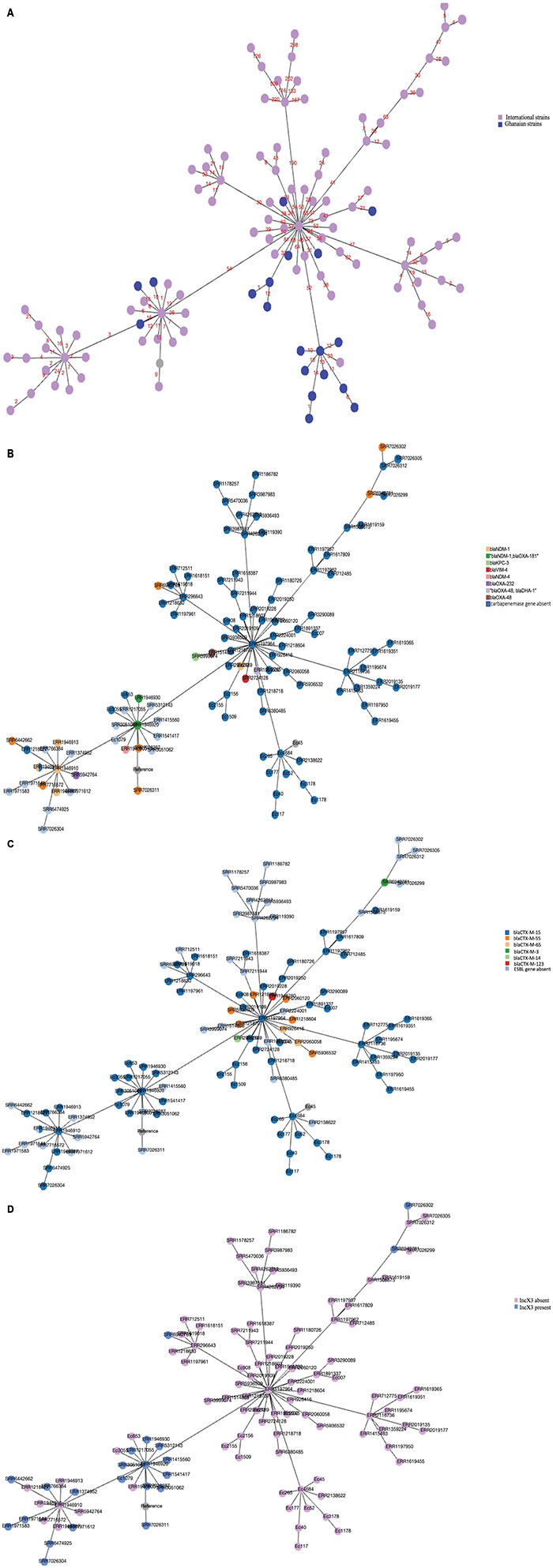
**(A)** Minimum spanning tree of core-gene alignment of 123 *E. coli* strains visualized with PHYLOViZ online. Twenty-one Ghanaian strains studied are colored in blue and 102 isolates from global collection are shown in pink color. Strains with similar sequences (Ec54 and Ec52), (Ec56 and Ec40) were represented by one node with slightly increased size. Numbers on the connecting lines denote number of locus variants. **(B)** Core genome alignment-based minimum spanning tree showing the presence of carbapenemase genes among strains. Gene presence and absence have been indicated by different colors as shown in the legend. Strains with similar sequences as detected by PHYLOviZ were represented by one node with slightly increased size. **(C)** Core genome alignment-based minimum spanning tree showing the distribution of ESBL genes among strains of various sub-lineages. Different colors depict different genes shown in the figure legend. Strains with similar sequences were represented by one node with slightly increased size. **(D)** Core genome alignment-based minimum spanning tree showing strains with IncX3 replicon. Strains with similar sequences were represented by one node with slightly increased size.

A minimum spanning tree was generated to assess the genetic relatedness or similarity between our ST410 strains amongst themselves, and with the other global strains. The analysis showed that the Ghanaians strains were genetically close to each other and although the Ghanaian strains were genetically distant from most of the global strains, there were a few strains that had a close genetic relationship with our Ghanaian strains, which suggests genetic similarities between the strains (Figure 4A). Antibiotic resistance genes were found in all sub-lineages but A/H53 and B1/H24 (Figures 4B,C). Strains in sub-lineage B4/H24RxC were mostly associated with IncX3 replicon (Figure 4D).

### Genetic Environment of bla_OXA–__181_

Hybrid assembly showed that strain Ec1079 contained one 4,807,180-bp chromosome and four circular plasmids ranging in sizes from 2 to 98 kb. *bla*_OXA–__181_ was found on an IncX3 plasmid designated pEc1079_3 (accession no. CP081309) and 51,479 bp in length.

**FIGURE 5:**
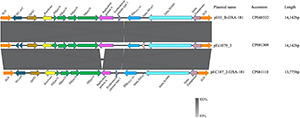
Linear comparison of the genetic environment of *bla*_OXA–181_. Sequences upstream and downstream of *bla*_OXA–181_ are shown with arrows, with the arrowheads indicating the direction of transcription. Truncated structures are indicated with denoted by “delta.” Different genes features are indicated by the different colors.

**FIGURE 6:**
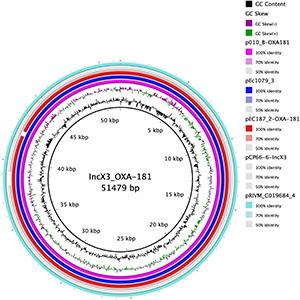
Comparison of plasmid pEc1079_3 with other reported IncX3 plasmids. Figure was drawn using BRIG comparison tool. BRIG considers sequences with 50–70% similarity as identical. Rings are arranged from inside out starting with the reference sequence p010_B-OXA181 as pink, plasmid pEc1079_3 as blue, plasmid pEc187_2-OXA-181 as red ring, plasmid pCP66-6-IncX3 as gray, and plasmid pRIVM_C019684_4 as turquoise ring. White spaces between rings show sequences that are present in the reference but absent in the query sequences and the degree of similarity.

We conducted a search of the *bla*_OXA–__181_ sequence region in the NCBI database using the nucleotide blast algorithm. The search produced 100% query cover and percentage identity with 22 other plasmid sequences in the database. The genetic structure of our *bla*_OXA–__181_ was compared with that of two of the plasmids, p010_B-OXA181 (accession no. CP048332) and pEC187_2-OXA-181 (accession no. CP061110) isolated from *E. coli* strains using Easyfig v2.2.2 (Sullivan et al., 2011). The genetic environment around *bla*_OXA–__181_ in pEc1079_3 was 100% homologous to that on p010_B-OXA-181, and almost to pEC187_2-OXA-181 except for a few deletions in the repA protein on pEC187_2-OXA-181. *bla*_OXA–__181_ was flanked by two copies of IS*26* composite transposons. It was also flanked by truncated IS*Ecp1*, IS*3000* and a partial Tn*3-*like transposase upstream. A replication protein A, IS*Kpn19* and a quinolone resistant gene, pentapeptide repeat protein *qnrS1* positioned between IS*2* ORF1 and a resolvase were found downstream (Figure 5). The whole length of the IncX3 plasmid pEc1079_3 from this study was also compared with that of four IncX3 plasmids reported in other parts of the world including a plasmid from Ghana (Figure 6). Comparison of the plasmids disclosed a highly conserved plasmid backbone. The results showed that the sequence of pEc1079_3 was highly identical to that of the reference plasmid recovered from an *E. coli* strain in Switzerland, p010_B-OXA181 (accession no. CP048332) and a *Klebsiella pneumoniae* isolate in the Netherlands, pRIVM_C019684_4 (accession no. CP068959). The sequences were also almost identical to, except for a few missing sequences in the IncX3 plasmid from Ghana, pEC187_2-OXA-181 (accession no. CP061110) and China, pCPP66-6_IncX3 (accession no. CP053726), both identified in *E. coli* strains.

## Discussion

Similar to ST131, ST410 has successfully been established as another ExPEC clone (Roer et al., 2018), with ESBL genes as the primary factors driving the spread of such resistance clones (Schaufler et al., 2016). Isolates that produce ESBL are, often, resistant to several other classes of antibiotics (Falgenhauer et al., 2016). In this study, all 21 ST410 isolates were confirmed as ESBL producers and exhibited a high rate (100%) of resistance to cefotaxime, ceftazidime, ciprofloxacin, piperacillin, sulfamethoxazole trimethoprim, and others. All these isolates harbored *bla*_CTX–M–__15_ which is the most widespread ESBL gene. *bla*_CTX–M–__15_–mediated resistance is a cause for concern because the genes are often carried on plasmids and can easily be passed on to other bacteria, increasing the resistant bacterial population, and in turn has a dire consequence on our therapeutic options.

Quinolone resistance in bacteria usually occurs when there are mutations in the sequences that code for QRDRs domains GyrA, ParC, and, less frequently, ParE (Solano-Gálvez et al., 2020). Consistent with what has been reported, we observed mutations in the mentioned QRDRs in all 21 isolates, in addition to fluoroquinolone resistance–mediating genes which consequently translated into the observed resistance.

The role of the IncF plasmid replicon types in the dissemination of resistance genes among Enterobacteriaceae has been widely discussed (Villa et al., 2010; Wang et al., 2013). In this study, IncF was the most common replicon type found, and, among *bla*_CTX–M–__15_ producing ESBLs, different IncF pMLST have been described with common ones being F2:A-: B-, F2:A1: B-, F22:A1:B20, F31:A4:B10, and F31:A4:B1. Some of these common sequence types have been identified to be prevalent in certain *E. coli* clones: F1:A2:B20 and F31:A1:B1 in ST405 and F2:A1:B- A in ST131 (Shin et al., 2012). A study by Nadimpalli et al. (2019) found ST410 *bla*_OXA–__181_- and *bla*_NDM–__5_-carrying carbapenemase encoding strains harboring pMLST F1:A1:B49. A *bla*_NDM–__1_-carrying *E. coli* ST410 was also found with an IncF plasmid with pMLST F1:A1:B49 in a study in Denmark (Overballe-Petersen et al., 2018). In agreement with this report, most isolates in this study harbored IncF replicon type of plasmids of six diverse pMLST. Among these, F31:A4:B1, F36:A4:B1, and F-:A1:B1, have been frequently reported. Notably, the *bla*_OXA–__181_- and *bla*_NDM–__1_-producing isolates harbored F1:A1:B49. The identification of this IncF replicon subtype and the other ones, F22:A4:B1 and F105:A4:B1, is evocative of an association with this clonal group.

In *E. coli* strains, adhesins, toxins, siderophores, lipopolysaccharide, capsule, and invasin are factors that regulate virulence (López-Banda et al., 2014) and are essential for survival in their host (Cyoia et al., 2015), with some virulence genes observed to be mostly present in specific clones (Devanga Ragupathi et al., 2020). The *ipf*A gene is reported to be peculiar to ST410 and encodes long polar fimbriae needed for adherence and intestinal colonization (Lloyd et al., 2012; Devanga Ragupathi et al., 2020). Although *ipf*A was not the most prevalent gene found, it was the second most common after *terC* and was found in over 80% of the isolates. For an ST that identifies with a non-pathogenic phylogroup, it is worthy to note also that each isolate carried at least two virulence genes and as many as eleven in a single strain (Supplementary Data Sheet 2).

Pangenome analysis of only the 21 Ghanaian strains showed a closed pangenome with higher number of core genes (4112) than accessory genes (2728). Our analysis of all the *E. coli* isolates pangenome revealed an open pangenome with 3681 core genes found in 98% and 10,308 accessory genes found in only a subset of the 123 strains. *E. coli* strains are known to remodel to suit their ecological niche as a result of horizontal DNA transfer and these transfers impact their accessory genome such that, the number of cloud genes increase with a rise in the number and diversity of isolates (Decano and Downing, 2019). However, going by the number of accessory genes in only the Ghanaian strains compared to that, with the global strains, we could deduce that Ghanaian strains are, to an extent, genetically diverse from the other strains.

Phylogenomic analysis of the isolates within a global context clustered some of our strains among ST410 subclades B3/H24Rx and B4/H24RxC described earlier (Roer et al., 2018). Saliently, a particular sub-lineage recently described (Feng et al., 2019) as closely related to B4/H24RxC appears to be creating a niche for itself in Ghana and perhaps, the African continent. A strain with an unspecified African origin included in a Chinese study (accession no. ERR1891337) was clustered closely with a strain from the United States among this sub-lineage (Feng et al., 2019). In our previous paper, one ST410 isolate identified was clustered among the same sub-lineage (Prah et al., 2021). From our analysis in this study, a good number (12/21) of our isolates also clustered with strains in the sub-lineage closely related to the globally disseminated group B4/H24RxC. However, the geographical information on the strains they clustered closely with was not available. In the future, further analysis would be helpful to accurately establish the sub-lineage of these strains and their associated characteristics. Three of the isolates were clustered among the globally disseminated B4/H24RxC. Even so, only one harbored a carbapenemase gene *bla*_OXA–__181_ gene and an IncX3 replicon, typical of members of this lineage (Roer et al., 2018). Molecular analyses have indicated potential clonal and horizontal spread of carbapenemases among inpatients in Iranian and Japanese hospitals (Ohno et al., 2017; Solgi et al., 2017). Carbapenems are the drug of choice for the treatment of severe infections due to ESBLs and, as such, resistance to these last line drugs is of great public health concern. Six of our isolates also clustered with strains in the B3/H24Rx sub-lineage. B3/H24Rx is reported to have emerged together with the B2/H24R around 1987, around the same time as the C1/H30R and C2/H30Rx clades in *E. coli* ST131, and subsequently evolved after it acquired *bla*_CTX–M–__15_ and an IncFII plasmid (Roer et al., 2018). As expected, all our six isolates within the sub-clade had *bla*_CTXM–__15_. Our *bla*_NDM–__1_ producing strain also clustered among sub-lineage B3/H24RxC.

Our observation on the minimum spanning tree (Figure 4A) further confirms that Ghanaian strains are diverse from the other strains included in the analysis. Figures 4B,C also showed that antibiotic resistant genes, thus, ESBL and carbapenemase genes were absent in strains in antibiotic-susceptible sub-lineages A/H53 and B1/H24 but were found in the resistant sub-lineages as described by Roer et al. (2018).

*bla*_OXA–__181_ has frequently been found on a 51-kb IncX3 plasmid flanked by two copies of the insertion sequence IS26 composite transposon (Liu et al., 2015; Nigg et al., 2019; Prah et al., 2021). From our analysis, we found *bla*_OXA–__181_ on a 51,479-bp IncX3 plasmid flanked by two copies of IS26 on either side, in the same orientation as a composite transposon, in agreement with these reports. IS26 has been reported to employ a replicative and self-targeted transposition to form an IS26 composite transposon entrapping resistance genes (Harmer and Hall, 2016). We could not demonstrate from this study that *bla*_OXA–__181_ was integrated into the IncX3 plasmid by this mechanism. However, given the available evidence, this phenomenon might not be far from the reality. Findings from previous studies provide an indication that IncX3 might be the vehicle mediating the dissemination of different carbapenemase alleles (Qin et al., 2016). The presence of *qnrS1* in the same region as *bla*_OXA–__181_ may have also selected for fluoroquinolone resistance in addition to the conferred resistance to carbapenems (Liu et al., 2015). IS*Ecp1* is usually found next to *bla*_OXA–__181_ and is suggested to play a role in its mobilization by misreading some sequences as its alternative right-hand inverted repeats (Liu et al., 2015). Plasmid comparison revealed a conserved plasmid backbone. The syntenic and conserved nature of the backbone of the IncX plasmid group plasmid has been cited as the reason for their stable evolution into successful subgroups as IncX3 (Johnson et al., 2012). The location and genetic environment of *bla*_NDM–__1_ have been described previously (Ayibieke et al., 2018).

ST410 is an international high-risk clone with resistance to multiple antibiotics. We resolved the phylogenomic relationship of 21 Ghanaian *E. coli* ST410 isolates with international strains. The analysis resulted in Ghanaian isolates distributed across the highly pathogenic sub-lineages B3/H24Rx, B4/H24RxC, and the closely related strains to B4/H24RxC, and these strains were associated with ESBLs and carbapenemase. This implies that ESBL and carbapenemase-resistant ST410 strains may be disseminating in these two hospitals. Haven been proven to be a globally distributed lineage with interspecies transmissibility and with effective transmission between patients, ST410 represents a public health threat and should therefore be monitored closely to prevent emergence of pan-drug resistant clones.

## Data Availability Statement

The datasets presented in this study can be found in online repositories. The names of the repository/repositories and accession number(s) can be found in the article/Supplementary Material.

## Ethics Statement

All protocols for the study were reviewed and approved by the Institutional Review Board of the Noguchi Memorial Institute for Medical Research, University of Ghana (FWA00001824) and Faculty of Medicine, Tokyo Medical and Dental University (M2017-208). Written informed consent was obtained from all participants of the study.

## Author Contributions

AAb and RS conceived the idea, designed the experiments, and supervised the study. SM, IP, AAy, and WS performed the experiments and analyzed the data. RS, TH, SI, and TS secured funding for the study. SM and RS wrote the original draft of the manuscript. All authors read and approved the final manuscript.

## Conflict of Interest

The authors declare that the research was conducted in the absence of any commercial or financial relationships that could be construed as a potential conflict of interest.

## Publisher’s Note

All claims expressed in this article are solely those of the authors and do not necessarily represent those of their affiliated organizations, or those of the publisher, the editors and the reviewers. Any product that may be evaluated in this article, or claim that may be made by its manufacturer, is not guaranteed or endorsed by the publisher.

## Footnotes

1
https://enterobase.warwick.ac.uk/

2
https://rast.nmpdr.org/

3
https://online.phyloviz.net/index

4
https://isfinder.biotoul.fr/

5
https://cge.cbs.dtu.dk/services/FimTyper/

6
https://cge.cbs.dtu.dk/services/PlasmidFinder/

7
https://cge.cbs.dtu.dk/services/pMLST/

8
https://cge.cbs.dtu.dk/services/VirulenceFinder/

## Supplementary Material

Supplementary Figure 1 Pangenome matrix of 21 *E. coli* strains from Ghana.

Supplementary Figure 2 Pie chart representation of core and accessory genes identified in the genomes of 21 *E. coli* strains from Ghana.
