# Supplementary figures and images for: Possible Dissemination of Escherichia coli Sequence Type 410 Closely Related to B4/H24RxC in Ghana
Source: Front Microbiol. 2021 Dec 1;12:770130. doi: 10.3389/fmicb.2021.770130 (PMC8672054; doi:10.3389/fmicb.2021.770130)

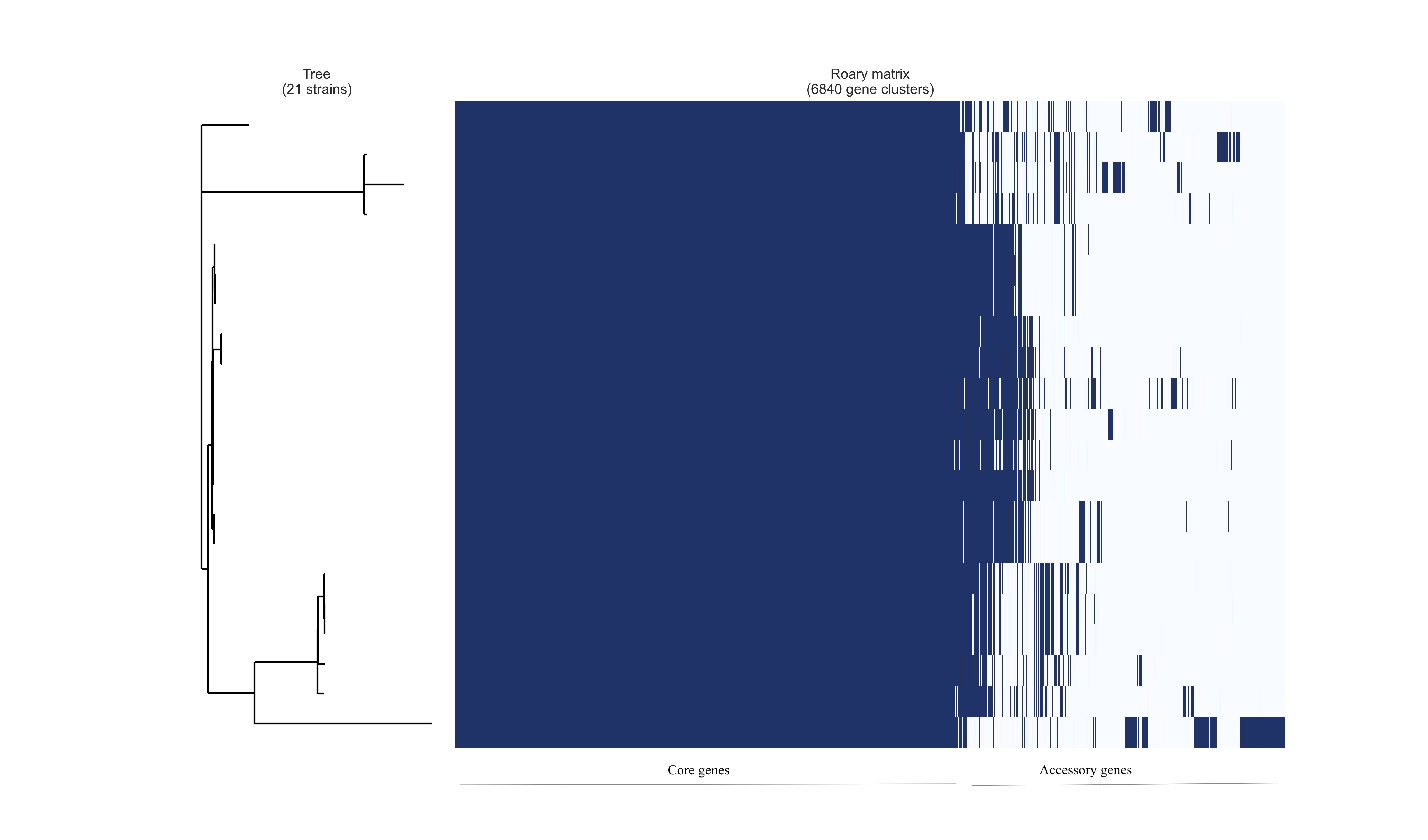

Supplement: Supplementary Figure 1 — Pangenome matrix of 21 E. coli strains from Ghana. [file Image_1.jpg]

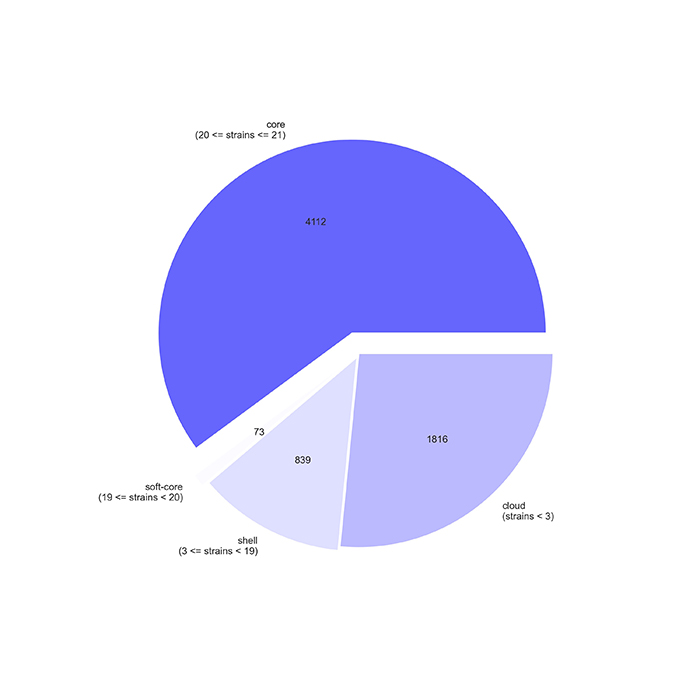

Supplement: Supplementary Figure 2 — Pie chart representation of core and accessory genes identified in the genomes of 21 E. coli strains from Ghana. [file Image_2.JPEG]
